# Key factors associated with nurse retention and how they work: A mixed-methods study

**DOI:** 10.1016/j.ijnsa.2026.100480

**Published:** 2026-01-07

**Authors:** Neeltje de Vries, Peter de Winter, Sanne Drost-Goossens, Hester Vermeulen, Catharina van Oostveen

**Affiliations:** a IQ Health Science Department, Radboud University Medical Centre, Nijmegen, the Netherlands; b Department of Internal Medicine, Spaarne Gasthuis Hospital, Haarlem and Hoofddorp, the Netherlands; c Spaarne Gasthuis Academy, Spaarne Gasthuis Hospital, Haarlem and Hoofddorp, the Netherlands; d Department of Paediatrics, Spaarne Gasthuis, Haarlem and Hoofddorp, the Netherlands; e Leuven Child and Health Institute, KU Leuven, Leuven, Belgium; f Department of Development and Regeneration, KU Leuven, Leuven, Belgium; g Department of Obstetrics and Gynecology, Spaarne Gasthuis Hospital, Haarlem and Hoofddorp, the Netherlands; h HAN University of Applied Sciences, School of Health Studies, Nijmegen, the Netherlands; i Erasmus School of Health Policy & Management, Erasmus University Rotterdam, Rotterdam, the Netherlands; j Amphia Research Centre, Amphia Hospital, Breda, the Netherlands

**Keywords:** Nursing staff, Hospital [MeSH], Personnel retention [MeSH], Factor analysis [MeSH], Q methodology, Nursing administration research [MeSH]

## Abstract

**Background:**

The global nursing shortage poses a critical challenge to healthcare systems. The World Health Organization projects a shortage of 4.5 million nurses by 2030. Contributing factors include an aging workforce and an increasing rate of nurse turnover, driven by high workloads, limited development opportunities, and a lack of managerial support. Intentions to leave predict actual departure and threaten the quality of care, increase patient mortality, and impose high replacement costs. While retention strategies exist, many are not tailored to nurses because nurses’ subjective experiences and perspectives are lacking, potentially limiting their effectiveness. Engaging nurses in designing these interventions may enhance their relevance and impact.

**Objectives:**

(i) Identify the key factors associated with nurses’ retention in hospitals, and (ii) provide an in-depth understanding of why nurses perceive these factors as critical in shaping their retention.

**Design:**

A mixed-methods approach, utilizing Q-methodology and semi-structured interviews, was employed.

**Setting:**

Dutch hospitals.

**Participants:**

A diverse group of 26 practicing and three former nurses.

**Methods:**

Participants ranked 58 statements in response to the prompt: “*I am willing to continue working as a nurse within the organization if…*” Consecutively, individual interviews were conducted to provide further insight into the reasoning behind nurses’ choices. Quantitative data were analyzed using centroid factor analysis with varimax rotation to identify shared viewpoints. Qualitative interview data were analyzed using the rigorous and accelerated data reduction method to deepen the understanding of the factors shaping nurses’ retention.

**Results:**

A total of 29 Q-sorts and interviews were analyzed. This resulted in three factors: (A) ‘Challenging work and inclusive positioning for high-quality care’, (B) ’Room to excel in nursing roles’, and (C) ‘Being seen, heard, and valued’. Together, these perceptions shaped participants’ intention to stay. Additionally, a common pattern across all factors was participants’ perception of being structurally unheard.

**Conclusions:**

A multifaceted interplay of factors influenced nurse retention. While meaningful and challenging tasks were essential, professional recognition, support systems, and growth opportunities were equally important. A systemic approach that addresses these various dimensions may be crucial for enhancing retention rates, maintaining a high-quality of patient care, and ultimately mitigating the impact of the nursing shortage in healthcare organizations. Nurses may play a crucial role in designing and implementing retention strategies. Their input is not just valuable but most likely necessary for the relevance and effectiveness of these strategies.

**What is already known**

• Nurse retention is a growing global concern with significant implications for organizational stability and the quality of patient care.
• Factors like workload, professional development, and managerial support are known to impact nurses’ job satisfaction and turnover intentions.
• It remains unclear whether existing retention strategies align with nurses’ perceptions, as many strategies are management-driven, potentially limiting their relevance and effectiveness.

Alt-text: Unlabelled box

**What this paper adds**

• Three interrelated factors were associated with nurses’ retention in hospitals: ‘challenging work and inclusive positioning for high-quality care’, ’room to excel in nursing roles’, and ‘being seen, heard, and valued’.
• Nurses felt structurally unheard at all organizational levels, a problem reinforced by the persistent neglect of well-documented retention factors in practice.
• To enhance the working environment for nurses and achieve long-term improvements in nurse retention a systemic, multi-level strategy may be required.

Alt-text: Unlabelled box

## 1 Introduction

The global shortage of healthcare personnel poses a significant worldwide challenge (Boniol et al., 2022). The World Health Organization estimates a shortage of 4.5 million nurses by 2030 (Boniol et al., 2022), a critical concern given nurses' pivotal role in healthcare. The aging nursing workforce contributes to this shortage, with many nurses nearing retirement age (White et al., 2025). For instance, 17 % of the nurses in the United States and Europe are expected to retire within the next decade (World Health Organization., 2020). Many nurses also consider leaving the profession due to high workloads, limited opportunities for professional development, and a lack of managerial support (De Vries et al., 2023a; Enea et al., 2024). In Europe, the percentage of nurses planning to leave the workforce ranges from 12.6 % in Belgium (Enea et al., 2024) to 30 % in Italy (Catania et al., 2024). Asian countries face similar rates, with 22.8 % of South Korean nurses having expressed their intention to leave the profession (Ki and Choi-Kwon, 2022). Because turnover intentions have been identified as a prerequisite and predictor of actually leaving the workforce (Halter et al., 2017; Hayes et al., 2006), these are concerning signs.

Retaining nurses is essential for maintaining high-quality patient care, as nurse turnover is linked to adverse outcomes (Bae, 2022), including higher mortality rates (Aiken et al., 2014; Catania et al., 2024), increased medical errors (O’Brien-Pallas et al., 2010; Winter et al., 2020), and patient dissatisfaction (De Simone et al., 2018). Additionally, the loss of nurses reduces the capacity to train nursing students in practice due to a lack of available supervision, hindering the education and practical training of a new generation, resulting in a limited internship availability for students and contributing further to workforce shortages. Furthermore, the average financial cost of replacing a nurse varied from $21,514 to $88,000 (Bae, 2022), illustrating the significant financial burden of recruitment on healthcare organizations. Hence, investing in the implementation of an effective retention strategy to sustain the nursing workforce is crucial.

Organizational conditions like reasonable workload, adequate resources, sufficient staffing ratios, and career development opportunities are pivotal to nurse retention (AbdELhay et al., 2025; Adams et al., 2021; Pressley and Garside, 2023). However, the absence of these conditions contributes to stress and burnout symptoms, which in turn negatively impact retention (Bahlman-van Ooijen et al., 2023; Enea et al., 2024). For example, the lack of sufficient staffing and protective equipment during the COVID-19 pandemic increased stress and led to higher turnover intentions (Dinçer and Altay, 2025; Enea et al., 2024; Farahani et al., 2024). The overall work environment, shaped by these organizational conditions, has significantly impacted nurse retention (Marufu et al., 2021). Previous researchers have consistently shown that a supportive and positive work environment is associated with higher job satisfaction and higher retention rates (Tan et al., 2024; Wan et al., 2018). Hence, improving nurses’ work environment and fostering a supportive culture are considered effective strategies to retain nurses (Pressley and Garside, 2023). Support from management, direct colleagues, and other disciplines positively impact retention (AbdELhay et al., 2025; Farahani et al., 2024; Marufu et al., 2021). Moreover, transformational leadership has been identified as an effective approach to fostering such environments, as it promotes engagement and professional recognition, ultimately enhancing nurse retention (AbdELhay et al., 2025).

In a recent study in the Netherlands, 24 % of the nurses intended to leave their hospital (van Kraaij et al., 2025), while 19.2 % intended to leave the profession (Enea et al., 2024). Dutch nurses emphasized the importance of alignment between their professional roles and personal values and described how constructive collaboration within an inspiring and safe team climate reinforced their motivation to remain in the profession (Seller-Boersma et al., 2023). However, a high workload and feelings of isolation due to staff shortages led to frustration and insecurity, particularly among novice nurses, undermining their sense of belonging (Kox et al., 2020). Seller-Boersma et al. (2023) emphasized the importance for organizational support in creating personalized career pathways and nurses’ opportunities to participate in shared decision-making, which contribute to a sense of recognition, autonomy, and professional fulfillment.

Several interventions have been developed to address nurse retention. In a recent review, De Vries et al. (2023b) identified several retention strategies, including onboarding programs to bridge the gap between education and clinical practice for novice nurses, communication strategies to enhance work relationships, and stress-coping interventions to support well-being. However, the long-term effect of these interventions remains unknown. Moreover, it is often unclear whether these interventions were designed with nurses in mind or based on their actual experiences (De Vries et al., 2023b). This poses a risk of disconnecting interventions from the daily realities of nursing practice, potentially limiting their effectiveness (Bamford et al., 2013). Seller-Boersma et al. (2023) emphasized this by highlighting that retention strategies must align with the nurses’ values, motivations, and experiences. Moreover, van Kraaij et al. (2025) described the importance of nurses’ autonomy in enhancing their work environment. Nurses’ involvement in designing and implementing retention strategies results in more credible and appropriate interventions (Brook et al., 2020; Robinson et al., 2022) because nurses are better positioned to address the underlying causes of their intention to leave the organization or profession. Furthermore, engaging nurses in such strategies makes them feel valued for their contributions (Brook et al., 2020). These factors have previously been identified as critical in influencing decisions to stay in the profession (Boone et al., 2024; De Vries et al., 2023a; Enea et al., 2024). This suggests that improving retention requires not only identifying what factors matter but also understanding why these factors matter.

While previous researchers have primarily focused on identifying which factors influence retention, less is known about why these factors matter and how they work (Adams et al., 2021; De Vries et al., 2023a; Farahani et al., 2024; Marufu et al., 2021). Addressing this gap is critical for managers, human resource advisors, and policymakers, as such insight enables them to translate retention factors into actionable strategies. Hence, with this study, we aimed to (i) identify the key factors associated with nurses’ retention in hospitals and (ii) provide an in-depth understanding of why nurses perceived these factors as critical in shaping their retention. By providing these fundamental insights, we intended to identify associations that may contribute to developing targeted and evidence-based strategies to improve nurse retention and ensure the stability and quality of healthcare services.

## 2 Methods

### 2.1 Design

This mixed-methods cross-sectional study employed a convergent sequential design. Quantitative and qualitative data were collected simultaneously using Q-methodology and interviews and subsequently analyzed in sequence. In Q-methodology, participants ranked a set of statements about retention factors on a grid (Q-sort). A factor analysis was then performed to systematically capture nurses’ perspectives on retention (Watts and Stenner, 2012). To explore why nurses sorted the statements as they did and to gain a deeper understanding of their rationale, semi-structured interviews were conducted immediately after the ranking (Gallagher and Porock, 2010). This triangulation enabled a comprehensive understanding of nurse retention (Creswell and Plano Clark, 2017).

### 2.2 Materials

A comprehensive list of statements (Q-set) was developed to answer the following question: “*I am willing to continue working as a nurse within the organization if…*” This Q-set was based on literature describing determinants impacting the intention to stay or leave (De Vries et al., 2023a, 2023b; Enea et al., 2024). To ensure the clarity and focus of the statements, the Q-set was refined in three rounds. In the first round, three researchers (NdV, PdW, and CvO) reviewed the initial set of statements to identify and eliminate overlap. In round two, three randomly selected registered nurses (RNs) from a medical department in a teaching hospital assessed the Q-set for face validity (Watts and Stenner, 2012). In round three, the Q-set was further refined based on their feedback by NdV, PdW, and CvO. These rounds were repeated until saturation was reached, meaning that no new insights emerged, and no further modifications of the statements were necessary during subsequent rounds (Churruca et al., 2021). This iterative process resulted in a final Q-set of 58 statements, covering fifteen Ncategories (see Supplementary Material S1). These categories were derived from prior literature on retention factors (De Vries et al., 2023a, 2023b; Enea et al., 2024) and informed the clustering of related statements by the research team. The purpose of the categories was to provide participants with a clear overview of the content.

### 2.3 Setting and participants

All RNs working in clinical departments in all Dutch general hospitals were invited to participate in the study. To create a representative sample, we employed maximum variation sampling to include participants of different sexes, ages, educational levels, departments, and hospital types (Watts and Stenner, 2012). To enhance the quality of collected data, we also purposively included former nurses, capturing the perspectives of individuals who had already left the profession. This selection process was designed to prevent bias and ensure the applicability of the study’s findings to a broad range of nursing contexts. Recruitment occurred through various social media platforms, including LinkedIn, Instagram, and Facebook. Additionally, potential participants in the researchers’ network were informed via email. Participants received a detailed study description and an invitation for an online interview. Written consent was obtained upon enrolment and reviewed verbally before data collection started.

### 2.4 Sample size

Watts and Stenner (2012) suggest that Q-methodology studies should include 40 to 60 participants, although they argue that considerably fewer can also be sufficient. Other researchers suggest a 3:1 ratio of statements to participants (Webler et al., 2009), which would result in a sample size of approximately 20 participants for our study.

### 2.5 Participant characteristics

Thirty nurses (26 practicing and four who had left the profession) participated in the study. One former nurse’s data was excluded due to missing responses, resulting in 29 analyzed participants. Sample characteristics are included in Supplementary Material S2.

### 2.6 Data collection

Data were collected from January 2024 to March 2024 using Microsoft Teams’ audio and video recording feature (Microsoft, 2023). The Q-sorTouch (2026) web application facilitated the sorting of the Q-set.

**Fig. 1:**
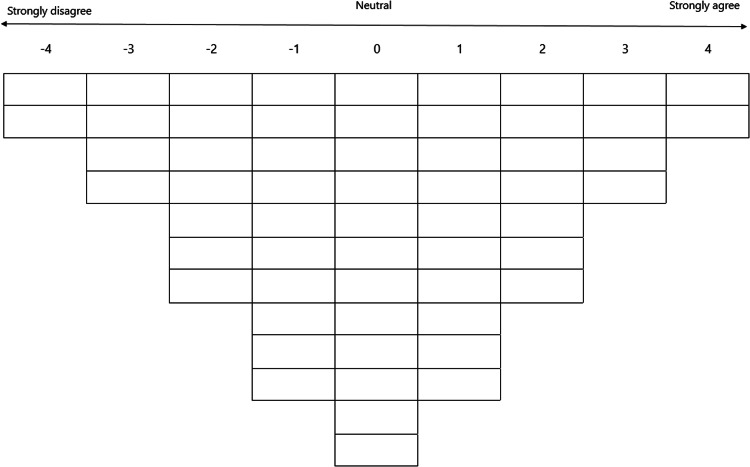
Q-sort table: A forced-choice frequency distribution table ranging from ‘strongly disagree’ to ‘strongly agree’ to sort the Q-set.

The data collection procedure included four steps. First, participants provided baseline demographic and professional characteristics, including sex, age, educational level, current position, current hospital, and years of work experience. Second, participants were presented with the phrase: “*I am willing to continue working as a nurse within the organization if…”.* They were instructed to rank the statements into three initial categories: agree, neutral, and disagree. Third, participants ranked the statements on a Q-sort table. This required them to distribute the statements across a quasi-normal, forced-choice frequency distribution table ranging from ‘strongly disagree’ to ‘strongly agree’. In our study, we structured the distribution on a 9-point Likert scale (see Fig. 1).

Lastly, after confirming the ranking, participants were interviewed individually, to explore the rationale behind their ranking choices. The interviews were guided by a pre-designed interview framework with open-ended questions (Gallagher and Porock, 2010) (see Supplementary Material S3). All interviews were transcribed using Microsoft Teams and carefully reviewed for accuracy prior to data analysis.

### 2.7 Data analysis

Descriptive statistics were generated using IBM SPSS Statistics, version 24.0. Categorical data were presented as counts and percentages, continuous non-normally distributed data as medians with interquartile ranges (IQRs).

To explore the shared viewpoints of the participants, a by-person factor analysis of the Q-sorts was conducted using PQ Method Software (Schmolck, 2014) with centroid factor analysis and varimax rotation (Watts and Stenner, 2012). This technique clusters participants with similar answers (Watts and Stenner, 2012). Each factor represents Q-sorts and, therefore, participants with a similar view or opinion on the intention to stay. To provide an in-depth understanding of why nurses perceived the elements of the statements critical to remaining in the current workplace, the interviews were analyzed using the rigorous and accelerated data reduction technique (Watkins, 2017).

#### 2.7.1 Quantitative data analysis

A factor was extracted if the following criteria were applicable (Hengeveld et al., 2021):

1) Horn’s parallel analysis: The number of factors to extract was determined by comparing the observed eigenvalues with the 95th percentile of the distribution of eigenvalues obtained from factor analyses of 1000 randomly generated datasets with the same sample size and number of variables (Horn, 1965).
2) Each factor should include two or more statistically significant factor loadings (*p* < 0.01). The factor loading greater or smaller than ± 2.58*(1/√(no of statements in Q-set) (Watts and Stenner, 2012) (i.e., ± 0.34 in this study) were considered significant.
3) ‘Humphrey’s rule’: The cross-product of the two highest loadings of the factor exceeds twice the standard error (SE) of the study (Watts and Stenner, 2012). The SE was calculated using 1/√(no. of statements in Q-set) (Brown, 1980), resulting in a SE of 0.13.

After the factor extraction, orthogonal rotation was applied using the varimax technique. Subsequently, the Q-sorts were assigned to a factor if they had a correlation of at least 0.6 on one factor and no >0.4 on any other factor (Watts and Stenner, 2012).

During the factor analysis stage, the shared viewpoints were consolidated into a single average Q-sort, referred to as a factor array. These factor arrays represent the composite rankings of statements. The factor arrays formed the foundation for interpreting the various factors (Watts and Stenner, 2012).

Additionally, consensus statements and distinguishing statements were examined. Consensus statements were statements whose rankings did not statistically significantly differ across any pair of factors (Watts and Stenner, 2012). Otherwise, distinguishing statements indicated that a particular factor ranked a statement statistically significantly (*p* < 0.01) differently than the other factors (Watts and Stenner, 2012).

For each factor, we constructed a crib sheet (Watts and Stenner, 2012) which included statements ranked at +4 and those ranked higher than other factors. Although it is common practice to include negative statements (−4) and those ranked lower than other factors in the crib sheet (Watts and Stenner, 2012), our focus was on the higher-ranked statements. These represented what statements participants perceived to be important with regard to retention.

#### 2.7.2 Qualitative data analysis

The qualitative analysis was performed after the quantitative analysis. This sequential integration ensured that the qualitative findings could be meaningfully interpreted alongside the quantitative results. In line with calls to capture the full diversity of voices in Q-method research (Watts and Stenner, 2012), we included interviews from both loaded and cross-loaded participants. These transcripts were analyzed for potential relevance to any factor identified in the quantitative analysis. Participants could meaningfully comment on each statements, regardless of their loading (Sneegas et al., 2021). Participants without any factor loading were excluded (Watts and Stenner, 2012).

We applied a three-phase rigorous and accelerated data reduction technique to analyze the interviews (Watkins, 2017). In phase one, NdV, SD, and NdB started reviewing the transcripts independently, extracting relevant quotes, and linking them to predefined factors and the crib sheets identified in the quantitative analysis using MAXQDA (VERBI Software, 2022). During in-person meetings, two pairs of researchers (NdV & SD and NdV & NdB) discussed the completeness of the selected quotes, their categorization under the relevant factors, and the assigned codes. Once consensus was reached, all data were compiled into a Phase-I table. This table was structured with columns corresponding to factors from the quantitative analysis. Previously agreed-upon quotes, codes, and categorizations were added to this table. An additional column was designated for quotes that did not align with the predefined factors, labelled as "other", and assigned descriptive keywords. CvO was available for collegial consultation to resolve any remaining uncertainties.

In the second phase, irrelevant or redundant information not needed to answer the research question was identified and removed by NdV and SD individually. This step was performed transparently, using the "track changes" function in Microsoft Word. Subsequently, an in-person meeting was held to resolve discrepancies, with input from CvO when needed. This collaborative effort resulted in the creation of the Phase-II table.

In the third phase, we analyzed the Phase-II table to identify the main themes related to nurses’ intention to stay within an organization. Three researchers (NdV, SD, CvO) independently and iteratively reviewed the organized text while keeping the research question in mind. Trends in the data and notable issues were discussed and summarized into codes. During consensus meetings, these codes were critically examined, refined, and consolidated into overarching themes. Supporting quotes from interviews were earmarked to illustrate the themes.

### 2.8 Rigor and reflexivity

Content validity of the Q-set was achieved by basing the statements on literature and refining them through interactive review by nursing experts and the research (Watts and Stenner, 2012). The multidisciplinary research team contributed relevant expertise to uphold methodological rigor (Akhtar-Danesh et al., 2008). NdV is an RN and PhD candidate trained in nursing science. PdW, a pediatrician and dean in medical sciences, contributed research experience across methodologies. SD is an RN and a clinical epidemiologist. She supported this study's methodological and practical aspects. HV, a nursing professor and clinical epidemiologist, provided oversight on epidemiological integrity. CvO, a senior researcher and former nurse, contributed her experience in qualitative research within this domain. NdB has a master’s degree in nursing science and is a non-practicing advanced nurse practitioner.

### 2.9 Ethical considerations

We conducted this study in accordance with the Declaration of Helsinki. It was approved by the local advisory board of Spaarne Gasthuis (number 2024.0039). Participants consented both verbally and in writing to participate and have their interviews recorded. Before giving consent, they were fully informed about the study and had the right to withdraw at any time.

During data collection, individual Q-sort data were temporarily stored in the secure QSorTouch web application (Q-sorTouch, 2026). After data collection was finished, the data were stored on a secure server and saved under the study identification number, in accordance with the rules and regulations of Spaarne Gasthuis Hospital. Interview data was exported to the secure server directly after finishing individual interviews. To ensure that no research data remained on external servers, both the web-based QSorTouch records and the Teams records were deleted. Pseudonymized data were analyzed in MaxQDA, accessible only to the study team via a secure institutional account.

## 3 Results

### 3.1 Quantitative results

Eight factors scored an eigenvalue >1.00. Five factors were excluded due to non-significant factor loadings, leaving three complying with Humphrey’s rule. Supplementary Material S4 outlines statements and factor arrays for all three factors. Factor A and B loaded both nine participants; factor C loaded two participants (see Table 1). One participant did not load significantly on any of the factors, while eight participants cross-loaded significantly on multiple factors.

**Table 1:** Participant characteristics and variables loaded on each factor.

	Factor A	Factor B	Factor C	Cross-loading
	(*n* = 9)	(*n* = 9)	(*n* = 2)	(*n* = 8)
Sex				
Male, *n* ( %)	2 (22.2)	0 (0)	1 (50)	1 (12.5)
Female, *n* ( %)	7 (77.8)	9 (100)	1 (50)	7 (87.5)
Age, Median [IQR]	42 [28–57.5]	37 [28–49]	33.5 [30–37]	38.5 [31–43.5]
Educational level				
Vocational degree, *n* ( %)	0 (0)	3 (33.3)	1 (50)	1 (12,5)
Bachelor’s degree, *n* ( %)	6 (66.7)	6 (66.7)	0 (0)	5 (62.5)
Master’s degree, *n* ( %)	3 (33.3)	0 (0)	1 (50)	2 (25)
Work experience in patient care (years), Median [IQR]				
	26 [7–38]	8 [5–32]	14 [7–21]	13 [7–24]
Work experience in current position (years), Median [IQR]				
	4 [2–9]	2 [2–5.5]	8.5 [1–16]	2 [1–5.5]
Function				
Nurse, *n* ( %)	7 (77.8)	9 (100)	2 (100)	7 (87.5)
Former nurse, *n* ( %)	2 (22.2)	0 (0)	0 (0)	1 (12.5)
Dual role, *n* ( %)	1 (11.1)	3 (33.3)	2 (100)	3 (37.5)
Hospital type				
Medical, *n* ( %)	1 (11.1)	2 (22.2)	0 (0)	2 (25)
Teaching, *n* ( %)	2 (22.2)	6 (66.7)	1 (50)	5 (62.5)
Academic, *n* ( %)	4 (44.4)	1 (11.1)	1 (50)	0 (0)
Other, *n* ( %)	2 (22.2)	0 (0)	0 (0)	1 (12.5)
Ward				
Medical, *n* ( %)	1 (11.1)	2 (22.2)	0 (0)	3 (37.5)
Surgical, *n* ( %)	2 (22.2)	0 (0)	0 (0)	1 (12.5)
Acute, *n* ( %)	2 (22.2)	2 (22.2)	2 (100)	2 (25)
Mixed, *n* ( %)	1 (11.1)	5 (55.6)	0 (0)	1 (12.5)
Other, *n* ( %)	3 (33.3)	0 (0)	0 (0)	1 (12.5)

*n* = subsample size, IQR = Inter Quartile Range, % = percentage.

Participants who loaded on factor A had more work experience in patient care compared to those loading on other factors, and most of them worked in an academic hospital. Most participants who loaded on Factor B were vocationally trained and worked in teaching hospitals. Notably, 37.5 % of the cross-loaded participants were employed in dual roles.

Factor A had an eigenvalue of 6.3 and explained 22 % of the total 45 % study variance. Focusing on the high-ranked crib sheet, factor A was characterized by five distinguishing statements (see Table 2). The factor represented ‘Challenging work and inclusive positioning for high quality care’.

**Table 2:** Crib sheet of high-ranked statements for factor A: Challenging work and inclusive positioning for high-quality care.

#	Statement	Factor array
36	There are opportunities for professional development	4
43	High-quality care can be provided	4*
58	Nurses and physicians work well together	3
42	I can provide safe patient care	3
19	There is a nurse scientist present in the organization	2*
37	I have the freedom to shape my own work	2
44	Clinical reasoning plays a crucial role in patient care	2*
45	There is a focus on evidence-based practice	2*
55	Communication among colleagues is effective	2
18	I am involved in policy decisions	1
13	The tasks I perform are diverse	1*
17	I am kept informed about changes within the organization	0
57	There is a collaborative atmosphere within the care network	0

⁎ Distinguishing statements *p* < 0.01.

Factor B had an eigenvalue of 4.6 and explained 16 % of the study variance. Defined as ‘Room to excel in nursing roles’, this factor included seven distinguishing statements and two consensus statements among its high-ranked statements in the crib sheet (see Table 3).

**Table 3:** Crib sheet of high-ranked statements for factor B: Room to excel in nursing roles*.*

#	Statement	Factor array
49	There is a good work-life balance	4*
52	*There is a positive work environment*	4
4	There is flexibility in my schedule	3*
25	*I can provide the necessary and desired patient care*	3
31	There is sufficient qualified staff to complete the work	3
47	I feel comfortable asking for help when needed	3*
5	There are sufficient medical supplies available for my daily tasks	2*
6	The ICT system functions well and provides adequate support	2
27	The organization addresses workload concerns	2
10	I have access to protocols and guidelines	1*
21	I receive a higher salary	1
26	I experience little negative stress from my work situation	1*
30	I still have energy left after work	1
3	I can do shift work	0*

⁎ Distinguishing statements *p* < 0.01; Consensus statements are given in italics.

Factor C had an eigenvalue of 1.9 and explained 7 % of the study variance. Described as ‘Being seen, heard and valued’, it included five distinguishing statements and six consensus statements in the high-ranked crib sheet.

Table 4.

**Table 4:** Crib sheet of high-ranked statements for factor C: Being seen, heard and valued*.*

#	Statement	Factor array
22	My work effort and workplace rewards are balanced	4*
36	There are opportunities for professional development	4
14	I can take on a dual role	3
24	*My work matches my level of education*	3
40	*I feel supported by my supervisor*	3
8	There are sufficient workspaces available	2*
9	The hospital is easily accessible from my home	2*
16	The organization values input from nurses (.e.g. through a nursing advisory council)	2
53	I can rely on my colleagues during difficult situations	2
15	The organization celebrates successes	1*
38	*My supervisor demonstrates strong leadership*	1
51	*The personal health of staff is prioritized*	1
20	*The organization practices sustainability in its use of resources and materials*	0
46	I have access to a buddy or mentor	0*
23	My work is appropriate for my age	−1
12	*Care assistants are present to support nurses*	−1

⁎ Distinguishing statements *p* < 0.01; Consensus statements are given in italics.

The correlation between the factor scores ranged from low to moderate. The lowest correlation occurred between factor B and factor C (*r* = 0.34), indicating that these two perspectives were the most distinct. The highest correlation was found between factor A and factor B (*r* = 0.45), indicating that these two perspectives have the most in common.

### 3.2 Qualitative results

Regarding the factor ‘Challenging work and inclusive positioning for high-quality care’, participants expressed the need for challenging and meaningful engagement in their professional roles within the organization in order to maintain high-quality care. They also emphasized the importance of being involved in the decision-making process. Regarding the factor ‘Room to excel in nursing roles’, participants emphasized their need for autonomy in how they could provide the best nursing care and maintain a good work-life balance. Furthermore, participants expressed the need to be trusted by the organization for their expertise. Regarding the factor ‘Being seen, heard and valued’, participants underscored the need for a workplace where they were recognized for their expertise and felt valued. However, they often experienced that their concerns were overlooked. These three factors revealed a common pattern across all factors: even though participants regularly expressed their needs, they felt structurally unheard.

While participants reflected on the selected and ranked Q-sorts, they gained a deeper understanding of the three factors identified through factor analysis. Additionally, their reflections also included reasons for staying or leaving the organization, as well as suggestions for how organizations could prevent them from leaving (Supplementary Material S5).

#### 3.2.1 Factor A: challenging work and inclusive positioning for high-quality care

Distinguishing for this factor was its strong focus on high-quality care through evidence-based practice, clinical reasoning, and the inclusion of nurse scientists within the organization. Participants expressed a desire for a stimulating work environment tailored to their specific professional needs and offering opportunities for growth and meaningful engagement in their professional roles. Challenging roles spanned clinical practice, management, and policymaking. Moreover, participants emphasized the need to be valued contributors of care teams and in the decision-making processes in maintaining high-quality care.

Participants considered their work challenging when they could engage in a diverse range of tasks that aligned with their educational background, thinking level, or career aspirations. This included taking on extra responsibilities and continually expanding professional knowledge. A key element was the preference for an autonomous nursing domain, operating alongside the medical domain in a more horizontal rather than hierarchical structure. This would allow participants to apply their expertise directly to patient care. Participants emphasized the importance of effective collaboration with physicians, where both professions respect and integrate each other's expertise to ensure the highest quality of care:

“Because you have to rely on each other, (…) it is important that you work as equals. Each with a different role. Not like in the past, when you worked under a doctor. No, you work alongside a doctor with your own expertise, and you also value each other for that.” - ID20

Participants stated that integrating clinical reasoning and evidence-based practice could further enhance their professional autonomy and their role in clinical decision-making processes and vice versa, contributing to greater job satisfaction. Conversely, when they were unable to fully utilize their expertise, they felt undervalued in their professional competence and reduced to executors of medical treatment policy, leading to decreased job satisfaction, engagement, and retention.

Moreover, participants expressed a strong desire to be actively involved in decision-making processes concerning their professional role within the organization rather than being presented with unilateral directives. They emphasized that professional advancement requires appropriate representation at all organizational levels. To achieve this, participants advocated for nursing advisory councils and nursing management positions to ensure that nurses' perspectives were included in hospital governance:

“We now have a nursing board alongside the medical board. And, well, that didn’t happen overnight. But it is essential. In the entire organization, the nursing group now has a seat at the executive table, alongside medical specialists. Without that, I wouldn’t be able to work in an organization anymore.” – ID20

Such inclusive positioning fostered participants’ professional development opportunities. Furthermore, engaging in dual roles, such as combining clinical practice with research or policy-oriented roles, was seen by participants as a valuable opportunity to contribute to quality improvement and healthcare innovation. However, such roles were not always standardly available in their work setting, which encouraged them to pioneer in creating these roles:

“(…) I am really grateful that I was eventually allowed to conduct research there and truly pioneer as a nursing scientist because that role did not exist within the department. I was able to accomplish some incredible things there.” – ID9

The lack of these dual roles prompted participants to discuss the development of specialized roles tailored to their personal expertise and career aspirations with their managers.

#### 3.2.2 Factor B: room to excel in nursing roles

Participants consistently emphasized the importance of room to excel in their nursing roles. This factor highlighted their need for autonomy and access to resources, including time, supplies, and equipment, to deliver high-quality nursing care and maintain a healthy work-life balance. Moreover, they wanted their organizations to trust their expertise.

The participants explained that having autonomy in managing a good work-life balance was pivotal for sustaining both their performance and their well-being. Many of them worked rotating shifts. While some appreciated the flexibility this offered, others found that rigid scheduling conflicted with their personal responsibilities, such as childcare or caregiving. Additionally, shift work often required more recovery time than was accounted for in the scheduling process:

“Precisely because I think it's a demanding profession, demanding in the sense of working shifts, for example. To illustrate this from my own experience, I had two night shifts on Friday and Saturday. Then, on Monday, I was supposed to work for the Nursing Advisory Council. Technically, that's not doable, but you also have obligations to the department. So, you think, ‘Okay, I'll just do it.’ So, you finish your night shift on Sunday and then on Monday at 8:30 AM, you're present at the office of the Nursing Advisory Council. You know, that’s not okay” – ID28

Therefore, participants advocated for realistic scheduling that considers nurses’ biological rhythms and individual needs for recovery time.

Self-scheduling was mentioned as a method to increase flexibility and autonomy. However, some participants noted that self-scheduling was ineffective when final rosters were altered by management, creating a false sense of control and leading to additional frustration:

"You go through the effort of self-scheduling, yet you don't get the shifts you requested. No, it makes no difference at all because, in the end, someone else still decides. So, I spend time planning when I want to work. Then, let's say, the real scheduler gets involved, and when I receive my final schedule, it's completely different. In the end, I still have to work on the days I didn't want to." – ID7

Participants expressed the need to provide the necessary and, according to their own standards, desired level of patient care. A balanced workload allowed them to engage in challenging and valued activities, such as quality improvement and evidence-based practice, which contributed to achieving their standard of quality care. A balanced workload, achieved by the availability of sufficient and efficient supplies, equipment, or technological systems, particularly outside standard office hours, enabled participants to fully engage in these activities. These elements lacking, increased stress, intensified the effect of high workload and limited their ability to excel in their roles:

“Having sufficient, well-functioning equipment is crucial. I currently work in a hospital that is very old and falling apart. That makes it difficult to carry out our work.” – ID22

Participants also emphasized the importance of up-to-date protocols and guidelines. Working in an organization that prioritizes current standards fosters feelings of safety and confidence:

“I once worked in a department where the protocols were not up to date. An external company had been hired to update them, but they couldn’t do it quickly enough. That created an extremely unsafe feeling because you have no idea whether you are still following the correct procedures (…). It was really uncomfortable. Sometimes I would go home thinking, ‘I’m not sure if I did everything right today.’” – ID25

Participants valued a work environment that recognized their professional expertise. While documentation requirements aimed to improve and monitor quality of care, participants highlighted the importance of balancing administrative duties, with, direct patient care. Overregulation in administrative tasks was perceived as undermining their professional judgment and autonomy. Therefore, participants argued that their clinical expertise should be trusted by board members, managers, and physicians, advocating for greater decision-making power in documentation and monitoring processes:

“The child abuse registration is now mandatory for every patient, appearing as a pop-up. Don't get me wrong, it's very good that we’re focused on this. You do need occasional reminders. But as an ER [Emergency Room] nurse, it’s second nature to me—when I see a child, I immediately think, 'Does this story add up?' If I see a spiral fracture, for example, alarm bells go off for all of us. That’s suspicious. However, now, this pop-up appears for every patient, and if you don’t fill it out, the patient can't be discharged. So, at the end of the day, someone is stuck in Electronic Patient Record filling out all these checklists for everyone” - ID14

#### 3.2.3 Factor C: being seen, heard, and valued

Distinctive for this factor was the focus on balanced workplace rewards, sufficient workspaces, accessibility of the hospital, formal recognition, and mentorship. Although valued across factors, participants valued professional development, appropriate work concerning educational levels and supervisory support, in this factor those items stood out for its emphasis on feeling genuinely recognized and valued by colleagues, managers, and the organization.

Participants emphasized the importance of a positive work culture. Feeling part of the team dynamics, sharing successes and social activities, and having space for humor, respect, and open communication fostered job satisfaction and resilience. In challenging situations, a psychologically safe environment enabled nurses to discuss difficult experiences openly without fear of judgment:

“For me, it is really important to work in a positive team where I feel comfortable with my colleagues. That trust allows me to provide the quality of care I want and to rely on my colleagues when needed.” – ID15

Participants highlighted the pivotal role of senior nurses and team leaders in shaping and maintaining this culture. Their leadership, through serving as role models or by mentoring less experienced staff, was seen as essential. This leadership was also seen as instrumental in creating the conditions necessary for professional development. Participants expressed that protected time for additional responsibilities was both a prerequisite for professional growth and an expression of appreciation and trust in their professional potential. This facilitated protected time reinforced their sense of being valued.

At the organizational level, participants highlighted that beyond a market-conform salary, non-financial benefits contributed significantly to feeling valued. A welcoming culture was reflected in practical measures like hospital accessibility, free parking, and meal provisions during evening and night shifts:

“But then I thought, this is absurd. A 16-year-old with a part-time job gets to pick €10 worth of products per shift from the store, yet here, during an evening shift, there is not even a place to get a hot meal. There’s no staff restaurant where you can go, even if you have to pay a small amount. It is really shocking that my 16-year-old son is better taken care of at his side job than we are in our permanent roles.” – ID24

Moreover, having essential resources readily available in their department, such as medical equipment or the presence of a care assistant to alleviate the workload, served as an important indicator of recognition. Participants also perceived the organization’s responsiveness to their requests, such as the prompt repair of essential medical resources, as a demonstration of being valued within their workplace.

An organization’s willingness to recognize and act on nurses’ contributions to work processes, efficiency improvements, and policymaking was perceived as valued. Participants expressed frustration when they encountered persistent resistance within the organization when sharing their expertise and professional insights:

"I think no, if you’ve tried to propose something 20 times and are constantly met with rejection, at some point, you either give up on the profession or resign yourself to the situation." – ID5

Nurses perceived the establishment of formal decision-making structures, including nursing advisory councils and management positions, within the hospital as a sign of appreciation.

Participants desired a broader societal understanding of the significance of nursing work, as they experienced negative societal prejudices associated with being a nurse. While these perceptions were not cited as direct reasons for leaving the profession, given the strong intrinsic motivation participants had, they contributed to feelings of frustration and a general sense of being underappreciated. Moreover, participants emphasized that the negative image may affect the inflow of new professionals, as the current public narrative does little to reflect how meaningful, enjoyable, and honorable they found their work:

"That’s why every year, at the end of the year, I ask health insurers not to waste money on meaningless advertisements about choosing them, since health insurance is mandatory, just like breathing. Instead, they should use that money to promote how incredibly rewarding it is to work in our profession and join us, showcasing everything one can achieve and contribute to being a nurse." – ID14

## 4 Discussion

### 4.1 Main findings

We identified three factors that were associated with nurses’ retention in hospitals: (A) C*hallenging work and inclusive positioning for high-quality care,* (B) *Room to excel in a nursing role*, and (C) *Being seen, heard, and valued.* The interviews illuminated the multifaceted dynamics behind these factors and provided insight into why they are crucial for participants' retention decisions. Nurse retention cannot be explained by individual factors alone but emerges from a complex interplay of interrelated elements of the work environment in which nurses operate (Adams et al., 2021; Efendi et al., 2019; Farahani et al., 2024).

We have highlighted the importance of challenging roles that align with nurses’ educational level, work experience, and expertise in retaining them within the organization, as discussed in factor A. Such alignment enables nurses to apply their competencies, expand their skills and grow within their profession, leading to retention (De Vries et al., 2023a; Kim and Shim, 2018). Participants working in academic hospitals and those with more experience particularly loaded on this factor, suggesting that experienced nurses may have a strong need for continuous professional development. In academic hospitals, opportunities for professional growth are possibly further supported by integration with academic initiatives, like university-led projects or research collaborations. Previous researchers underscored the importance of combining clinical practice with academic roles to enhance both intellectual stimulation and practical career advancement, eventually leading to retention (Galuska et al., 2023; van Oostveen et al., 2017). Nevertheless, even less experienced nurses may benefit from challenges tasks beyond routine care and opportunities to apply competencies (Kox et al., 2020). Providing challenging roles and tasks across career stages may be, therefore, essential to maintain engagement, foster growth, and support retention (Kox et al., 2020; Pressley and Garside, 2023).

While challenging roles matter, the finding of factor B indicated that nurses may also need opportunities to excel to remain in their positions. However, due to excessive workload, time constraints, or insufficient resources, they faced practical barriers and experienced stress linked to diminished personal and professional fulfilment (Brouwer et al., 2025). To mitigate this, organizations should consider ensuring role clarity and provide sufficient (resource) support. Participants described the availability of tools and protected time for professionally meaningful tasks as essential, echoing literature linking resource sufficiency to professional satisfaction (Hakvoort et al., 2022; Van der Heijden et al., 2019). Without these conditions, ambition may remain unfulfilled and retention may be jeopardized.

Autonomy in work-life balance emerged as a critical enabler of nurses’ well-being (Stimpfel et al., 2025) and a condition for excelling in nursing roles. For example, granting nurses autonomy over their schedules allows them to create a schedule that suits both their professional and personal lives. This likely explains why participants frequently mentioned self-scheduling. Brouwer et al. (2025) support this, emphasizing that fulfilling autonomy needs can buffer work demands and sustain energy and work-life balance. Ultimately, when nurses can balance their professional and personal responsibilities, they are better engaged in their roles, contributing to higher job satisfaction and lower turnover (Brouwer et al., 2025; Stimpfel et al., 2025).

In line with this need for stability and balance, participants framed financial compensation as a practical condition for sustaining both work and life. Rather than a sign of professional recognition (De Vries et al., 2023a), salary was described as essential to covering fixed costs like mortgages or insurance costs. This financial pressure has been found to contribute to emotional strain, reduced professional engagement, and limited investment in organizational goals (Almalki et al., 2012; Sibuea et al., 2024). Hence, inadequate compensation can drive attrition when it undermines nurses’ ability to maintain a stable and manageable life.

In contrast, age-appropriate work design was valued both for its practicality and as a sign of recognition. Tailoring roles and tasks to nurses’ capacities across life stages may foster retention by addressing their evolving needs (Uthaman et al., 2015; Van der Heijden et al., 2019). Considering the existing global shortage (Boniol et al., 2022) and the ageing nursing workforce (White et al., 2025), this highlights the importance of creating an environment where individual nurses feel valued, remaining engaged and committed (van Kraaij et al., 2024a).

This theme of being valued also strongly emerged in how participants talked about their professional identity, as stated in factor C. They expressed a strong desire for work environments where they were acknowledged as autonomous professionals vital for high-quality patient care (van Oostveen et al., 2015; Vermeulen and van Leeuwen, 2023). Moreover, a work environment with support from direct colleagues and management has been associated with increased morale, job satisfaction, and a lower intention to leave (Kaan Namal et al., 2024; Seller-Boersma et al., 2023). We have highlighted that nurses’ perceptions of not being heard extend beyond interactions with direct colleagues and manager, similar to other researchers (Kaan Namal et al., 2024; Seller-Boersma et al., 2023). Therefore, the presence of nursing advisory councils and strategic positions within their organizations is recommended (Heinen et al., 2013). Without these organizational structures, nurses have reported feeling professionally invisible, which could negatively affect staff morale and patient care (Felder et al., 2021).

This sense of invisibility reinforced the frustration participants often felt when they had limited influence in both clinical and organizational decision-making. Participants described situations where policies were changed without consulting frontline nursing staff, despite the direct impact on their daily work. This exclusion not only undermined their professional autonomy but also contributed to feelings of disempowerment. This finding aligns with Seller-Boersma et al. (2025), who found that a lack of decision-making power influenced nurses’ intention to leave. Hence, embedding nursing perspectives into policy and leadership structures may therefore be a necessity. Promoting leadership and decision-making authority (e.g., through nursing advisory boards and shared governance) could foster professional ownership and institutional responsiveness (Alshareef et al., 2020; Seller-Boersma et al., 2023).

The lack of such structural responsiveness was evident across all three study factors, revealing a common pattern: participants felt structurally unheard. Moreover, the key retention factors identified in our study have been repeatedly identified in previous research (Alshareef et al., 2020; Brouwer et al., 2025; De Vries et al., 2023a) but remain unaddressed by effective retention strategies. This can be seen as confirmation that nurses are not being heard structurally. Verhoeven et al. (2024) supported this by illustrating how communication patterns within hospital boards marginalize nursing perspectives, pointing to broader organizational neglect rather than isolated failures. Hence, the challenge is not merely improving individual job satisfaction to improve retention but reconfiguring the complex systemic work environment in which nurses operate. The complexity is particularly evident in how nurses’ ambitions for growth interact with systemic constraints. While participants expressed the desire for more challenging roles, their ambition was frequently met with frustration due to limited time, resources, and managerial support. Similar findings emerged for nurses working within rigid organizational structures (Brouwer et al., 2025; van Kraaij et al., 2024b).

Notably, the presence of cross-loaded participants indicates that there are additional nuance viewpoints beyond those captured by the three factors. Similarly, including both distinguishing and consensus statements in the high-ranked crib sheets illustrated the intertwined nature of different perspectives on retention. Furthermore, the consensus statements reinforce areas of overlap between viewpoints (Exel and Graaf, 2005), further highlighting the complexity of retention. This underscores that retention is not merely the result of individual elements but rather a complex interplay of interrelated elements of the broader work environment in which nurses operate. Hence, the three factors found in our study should not be viewed in isolation. As previous researchers have suggested, enhancing the work environment systemically rather than addressing isolated components is key to reducing turnover intention (Bloemhof et al., 2021; van Kraaij et al., 2025). Van Kraaij et al. (2025) further argue that nurses in strategic positions are vital for reforming nursing practice and breaking the cycle of structural silence. Operating at key positions, nurses can translate frontline experience into policy and respond more effectively to bureaucratic bottlenecks and enable faster and better-aligned implementation of changes (Stalter and Mota, 2018; van Kraaij et al., 2025), enhance work environment (van Kraaij et al., 2025), and potentially promote retention (van Kraaij et al., 2025).

### 4.2 Strengths and limitations

This mixed-method Q-study offered several strengths. First, using statements from existing literature ensures a strong theoretical foundation, enhancing credibility and relevance (Watts and Stenner, 2012). Second, the mixed-methods Q-study approach reveals distinct perspectives within nursing, offering valuable insights into diverse experiences and priorities of nurses (Gallagher and Porock, 2010; Watts and Stenner, 2012). Moreover, by employing a convergent parallel design, finding during the interpretation phase enabled a robust and nuanced analysis, thereby enhancing the validity of the conclusions through the comparison and contrast of the quantitative patterns with rich qualitative narratives (Creswell and Plano Clark, 2017).

Nonetheless, certain limitations should be acknowledged. Some participants reported difficulty ranking statements, viewing all as relevant to their retention decisions. This was anticipated, given that statements were derived from established literature. Therefore, lower-ranked statements should not be dismissed as unimportant but rather understood as foundational elements necessary for effective job performance. Moreover, participants’ ranking of statements did not always correspond precisely with how they described their perspectives in interviews, which can complicate the integration of quantitative and qualitative findings (Creswell and Plano Clark, 2017). However, this discrepancy is inherent to the methodology and can be mitigated through careful triangulation (Creswell and Plano Clark, 2017; Watts and Stenner, 2012).

Another limitation relates to the representativeness of factor C, which significantly loaded on only two Q-sorts. While this may suggest a lower general support for this viewpoint, factors defined by a small sample can yield meaningful insights. Moreover Q-methodology aims to identify different perspectives rather than provide a statistical representation (Akhtar-Danesh et al., 2008).

Furthermore, the study’s setting in Dutch hospitals and the high educational level of many participants may limit generalizability. However, many themes identified will be recognized by diversely educated nurses, also internationally.

### 4.3 Implications for practice and research

By sharing their views on retention, participants suggested various potential retention strategies. However, addressing the complexity of retention and the need for systemic strategies, may require multifaceted interventions at individual, organizational and health system levels.

Allowing nurses to manage work-life balance may be essential (Brouwer et al., 2025; Stimpfel et al., 2025). Additionally, both financial (fair salaries) and non-financial (mentorship programs, professional autonomy, working conditions) aspects should play a significant role in retention strategies (Almalki et al., 2012; Kaan Namal et al., 2024). At the individual level, nurses should be empowered to take leadership of their professional practice and assert their voice in everyday decision-making (Kaan Namal et al., 2024). Cultivating a workplace culture that values nurses’ participation can enhance agency, accountability, and organizational commitment (Kaan Namal et al., 2024; Seller-Boersma et al., 2023). At the organizational level, structures for shared governance, like nursing advisory councils and decision-making committees, can promote control over practice and autonomy, and integrate nursing more meaningfully into strategic planning (Seller-Boersma et al., 2023). Supportive and visible nursing leadership is essential to create a positive work environment enabling nurses to contribute to the decision making process, and fostering opportunities for them to grow into (strategic) leadership roles (Almalki et al., 2012; Sibuea et al., 2024; van Kraaij et al., 2024a). At the broader healthcare system level, sustainable retention strategies depend on formally recognizing nursing expertise in national policy and governance (van Oostveen and Vermeulen, 2017), including nursing representation in advisory and political roles (Seller-Boersma et al., 2023). Most importantly, management-led initiatives must acknowledge and reinforce the structural influence that nurses expect and aim to have.

Whereas we suggest existing interventions lack the necessary depth to tackle the multiple, interrelated factors that contribute to nurses’ retentions, future researchers should focus on designing, implementing, and evaluating the effectiveness of multifaceted systemic interventions (Seller-Boersma et al., 2023). Since nurses’ involvement is a known factor in the success of retention strategies (Brook et al., 2020; Robinson et al., 2022), participatory action research approaches offer a valuable framework for future research, enabling nurses to influence intervention design and implementation directly. Moreover, to study the effectiveness of the intervention, future researchers should include longitudinal study designs incorporating repeated measures to evaluate the intervention’s effect reliably.

## 5 Conclusions

Nurse retention in hospitals is shaped by a systemic interplay of interrelated elements of the nursing work environment. We identified three crucial, interconnected conditions for retention: the opportunity to engage in challenging, meaningful work with high-quality care; the room to grow and excel within the nursing role; and the experience of being genuinely seen, heard, and valued. We showed the interconnection of these factors was anchored in the experience of structurally being unheard. This may indicate that retention through isolated measures is unlikely to be effective; it requires systemic strategies that embed active listening to nurses’ voices into governance structures, strengthen their influence in decision-making processes, and respond meaningfully to their professional and personal needs. By addressing these factors in their interdependence, hospitals may be able to create conditions that enable nurses to remain working for their organizations.

## Funding sources

This study did not receive any specific grant from funding agencies in the public, commercial, or not-for-profit sectors.

## Declaration of generative AI and AI-assisted technologies in the writing process

During the preparation of this work, the author(s) used ChatGPT 4 for proofreading of the manuscript. After using this tool, the author(s) reviewed and edited the content as needed and take full responsibility for the content of the publication.

## CRediT authorship contribution statement

**Neeltje de Vries:** Writing – review & editing, Writing – original draft, Visualization, Supervision, Resources, Project administration, Methodology, Investigation, Formal analysis, Data curation, Conceptualization. **Peter de Winter:** Writing – review & editing, Validation, Supervision, Methodology, Conceptualization. **Sanne Drost-Goossens:** Writing – review & editing, Validation, Formal analysis. **Hester Vermeulen:** Writing – review & editing, Supervision, Methodology, Conceptualization. **Catharina van Oostveen:** Writing – review & editing, Visualization, Supervision, Methodology, Formal analysis, Conceptualization.

## Declaration of competing interest

The authors declare that they have no known competing financial interests or personal relationships that could have appeared to influence the work reported in this paper.

## Data Availability

The dataset is not publicly available due to the sensitive nature of the interviews and the inability to fully anonymize the data.

## Appendix Supplementary materials

Image, application 1
