# Supplementary material for: Key factors associated with nurse retention and how they work: A mixed-methods study
Source: Int J Nurs Stud Adv. 2026 Jan 7;10:100480. doi: 10.1016/j.ijnsa.2026.100480 (PMC12857392; doi:10.1016/j.ijnsa.2026.100480)
Supplement: Supplementary file 1 [file mmc1.docx]

# Supplementary Material S1. Statements Q-set.

|  | *I am willing to continue working as a nurse within the organization if…”* |
| --- | --- |
| Work flexibility | I am able to work part-time |
|  | I have the ability to create my own schedule |
|  | I can do shift work |
|  | There is flexibility in my schedule |
| Resource availability | There are sufficient medical supplies available for my daily tasks |
|  | The ICT-system functions well and provides adequate support |
|  | There is a therapy dog on the ward for staff to interact with |
|  | There are sufficient workspaces available |
|  | The hospital is easily accessible from my home |
|  | I have access to protocols and guidelines |
| Workplace amenities | There are designated areas for lunch |
|  | Care assistants are present to support nurses |
|  | I experience diversity in the tasks I perform |
|  | I can take on a dual role |
| Organizational support and engagement | The organization celebrates successes |
|  | The organization values input from nurses (e.g. through a nursing advisory council) |
|  | I am kept informed about changes within the organization |
|  | I am involved in policy decisions |
|  | There is a nurse scientist present in the organization |
|  | The organization practices sustainability in its use of resources and materials |
| Compensation and recognition | I receive a higher salary |
|  | My work effort and workplace rewards are balanced |
| Role suitability | My work is appropriate for my age |
|  | My work matches my level of education |
| Workload management | I can provide the necessary and desired patient care |
|  | I experience little negative stress from my work situation |
|  | The organization addresses workload concerns |
|  | The tasks required can be completed within the available time |
|  | I can take adequate breaks |
|  | I still have energy left after work |
|  | There is sufficient qualified staff to complete the work |
|  | I am not required to perform non-nursing tasks |
|  | The administrative burden is reduced |
| Professional role models | There are role models within the organization |
| Development and growth | There is room for personal development |
|  | There are opportunities for professional development |
|  | I have the freedom to shape my own work |
| Leadership and team dynamics | My supervisor demonstrates strong leadership |
|  | I feel like a valued member of a well-managed team |
|  | I feel supported by my supervisor |
|  | My supervisor is open and transparent about ongoing matters |
| Quality of patient care | I can provide safe patient care |
|  | High-quality care can be provided |
|  | Clinical reasoning plays a crucial role in patient care |
|  | There is a focus on evidence-based practice |
| Support systems | I have access to a buddy or mentor |
|  | I feel comfortable asking for help when needed |
| Diversity and inclusivity | Unacceptable behavior is addressed promptly |
| Health and resilience | There is a good work-life balance |
|  | Mental health issues are acknowledged and supported |
|  | The personal health of staff is prioritized |
| Teamwork and collaboration | There is a positive work environment |
|  | I can rely on my colleagues during difficult situations |
|  | Colleagues feel a responsibility to give and receive feedback within the team |
|  | Communication among colleagues is effective |
|  | I feel a sense of belonging within the team |
|  | There is a collaborative atmosphere within the care network |
|  | Nurses and physicians work well together |

# Supplementary Material S2. Sample characteristics and variables under study.

|  | Total *N*=29 |
| --- | --- |
| Sex  Male, *n* (%)  Female, *n* (%) | 4 (13.8)  25 (86.2) |
| Age, Median [IQR] | 38 [30-50] |
| Educational level  Vocational degree, *n* (%)  Bachelor’s degree, *n* (%)  Master’s degree, *n* (%) | 5 (17.2)  18 (62.1)  6 (20.7) |
| Work experience in patient care (years), Median [IQR] | 16.5 [7-29] |
| Work experience in current position (years), Median [IQR] | 2 [2-6] |
| Function  Nurse, *n* (%)  Former nurse, *n* (%) | 26 (89.7)  3 (10.3) |
| Dual role, *n* (%) | 10 (34.5) |
| Hospital type  General, *n* (%)  Teaching, *n* (%)  Academic, *n* (%) | 5 (17.2)  17 (58.6)  7 (24.1) |
| Department  Medical, *n* (%)  Surgical, *n* (%)  Acute, *n* (%)  Mixed, *n* (%)  Other, *n* (%) | 6 (20.7)  3 (10.3)  12 (41.4)  7 (24.1)  1 (3.4) |

*n = subsample size, IQR = Inter Quartile Range, % = percentage*

# Supplementary Material S3. Interview guide.

**Introduction**

- Explain: This research contribute to the development of an intervention aimed at retaining nursing staff within the hospital setting.
- Inform: This interview will take approximately one hour.
- Emphasize: There are no right or wrong answers in this interview; we are interested in your perspectives.
- Request consent to record the interview and use the data for my doctoral research (a consent form was included in the survey).

**Recording**

Start the recording in Microsoft Teams, including transcription.

**Opening Question (prior to sorting)**

Complete the phrase: "*I would like to continue working as a nurse within this organization if..."*

**Instructions for Q-Sorting**

The sorting of statements follows a structured methodology. There are 58 cards with statements that need to be sorted on the Q-grid. The Q-grid consists of nine columns, with the far-left column indicating “strongly disagree” and the far-right column indicating “strongly agree.”

- Sort the items into three piles: the left pile for “unimportant,” the right pile for “important,” and the middle pile for items about which you are unsure or neutral.
- Arrange the “important” pile onto the Q-grid.
- Arrange the “unimportant” pile onto the Q-grid.
- Arrange the “neutral” pile onto the Q-grid.
- Place items about which you are unsure in the column you find most suitable. It is permissible to adjust the placement of items already positioned.
- Review the arrangement of items and decide if you wish to make any changes.
- Following the sorting of items, a brief interview will take place.

**Q-Interview**

Engage the participant in a discussion about the items located in the two extreme columns of the Q-grid. Use open-ended prompts to encourage elaboration, such as:

- Could you explain why … is most important to you?
- Could you explain why … is least important to you?

Alternatively, inquire about an item that stands out to you.

Ensure that the following questions are also addressed:

- Could you explain the influence of item … on your decision to remain working as a nurse?
  - Item …
  - Item …
- Are there any additional factors that you believe are important for retaining you as a nurse (factors not included in the statements)?

**End of Recording**

Stop the recording in Microsoft Teams.

**Closing**

Ask the participant for feedback on the methodology and thank them for their participation.

# Supplementary Material S4. List of statements with factor arrays.

| I am willing to work as a nurse within the organization if: | Factor A | Factor B | Factor C |
| --- | --- | --- | --- |
| 1. *I am able to work part-time* | -2 | -2 | -3 |
| 1. I have the ability to create my own schedule | **-2*** | 2 | 2 |
| 1. I can do shift work | -2 | **0*** | -3 |
| 1. There is flexibility in my schedule | 0 | **3*** | -1 |
| 1. There are sufficient medical supplies available for my daily tasks | -1 | **2*** | -2 |
| 1. The ICT-system functions well and provides adequate support | 0 | 2 | 1 |
| 1. *There is a therapy dog on the ward for staff to interact with* | -4 | -4 | -4 |
| 1. There are sufficient workspaces available | -1 | -1 | **2*** |
| 1. The hospital is easily accessible from my home | **-3*** | **0*** | **2*** |
| 1. I have access to protocols and guidelines | 0 | **1*** | -1 |
| 1. *There are designated areas for lunch* | -3 | -3 | -4 |
| 1. *Care assistants are present to support nurses* | -2 | -2 | -1 |
| 1. The tasks I perform are diverse | **1*** | -1 | -2 |
| 1. I can take on a dual role | 1 | **-2*** | 3 |
| 1. The organization celebrates successes | -2 | -2 | **1*** |
| 1. The organization values input from nurses (e.g. through a nursing advisory council) | 1 | **0*** | 2 |
| 1. I am kept informed about changes within the organization | 0 | -1 | -1 |
| 1. I am involved in policy decisions | 1 | **-3*** | -1 |
| 1. There is a nurse scientist present in the organization | **2*** | -2 | 0 |
| 1. *The organization practices sustainability in its use of resources and materials* | -1 | -1 | 0 |
| 1. I receive a higher salary | -2 | 1 | 0 |
| 1. My work effort and workplace rewards are balanced | 1 | 1 | **4*** |
| 1. My work is appropriate for my age | **-3*** | -2 | -1 |
| 1. *My work matches my level of education* | 1 | 0 | 3 |
| 1. *I can provide the necessary and desired patient care* | 2 | 3 | 1 |
| 1. I experience little negative stress from my work situation | -1 | **1*** | **-2*** |
| 1. The organization addresses workload concerns | 0 | 2 | 1 |
| 1. *The tasks required can be completed within the available time* | 0 | 0 | -1 |
| 1. *I can take adequate breaks* | -1 | -1 | -2 |
| 1. I still have energy left after work | -2 | 1 | 0 |
| 1. There is sufficient qualified staff to complete the work | 1 | 3 | 2 |
| 1. *I am not required to perform non-nursing tasks* | -4 | -3 | -3 |
| 1. *The administrative burden is reduced* | -1 | 0 | 0 |
| 1. There are role models within the organization | -1 | **-3*** | -1 |
| 1. There is room for personal development | 3 | **0*** | 3 |
| 1. There are opportunities for professional development | 4 | **2*** | 4 |
| 1. I have the freedom to shape my own work | 2 | **-1*** | 1 |
| 1. *My supervisor demonstrates strong leadership* | 0 | 0 | 1 |
| 1. *I feel like a valued member of a well-managed team* | 0 | 0 | -1 |
| 1. *I feel supported by my supervisor* | 1 | 0 | 3 |
| 1. *My supervisor is open and transparent about ongoing matters* | 0 | -1 | 0 |
| 1. I can provide safe patient care | 3 | 2 | **-2*** |
| 1. High-quality care can be provided | **4*** | **1*** | **-3*** |
| 1. Clinical reasoning plays a crucial role in patient care | **2*** | -1 | 0 |
| 1. There is a focus on evidence-based practice | **2*** | -2 | -3 |
| 1. I have access to a buddy or mentor | **-3*** | **-4*** | **0*** |
| 1. I feel comfortable asking for help when needed | -1 | **3*** | -2 |
| 1. *Unacceptable behavior is addressed promptly* | 2 | 2 | 0 |
| 1. There is a good work-life balance | -1 | **4*** | 1 |
| 1. *Mental health issues are acknowledged and supported* | 0 | 0 | 0 |
| 1. *The personal health of staff is prioritized* | -1 | -1 | 1 |
| 1. *There is a positive work environment* | 3 | 4 | 2 |
| 1. I can rely on my colleagues during difficult situations | **0*** | 1 | 2 |
| 1. Colleagues feel a responsibility to give and receive feedback within the team | 1 | 0 | 1 |
| 1. Communication among colleagues is effective | 2 | 1 | **-1*** |
| 1. *I feel a sense of belonging within the team* | 1 | 1 | -1 |
| 1. There is a collaborative atmosphere within the care network | 0 | -1 | -2 |
| 1. Nurses and physicians work well together | 3 | 1 | **-2*** |

^*^ Distinguishing statement *p*<0.01

Consensus statements are given in italics

# Supplementary Material S5. Recommendations to prevent nurses from leaving the organization.

| Reasons for leaving the organization | Reasons for staying in the organization | | | Recommendations | |
| --- | --- | --- | --- | --- | --- |
| Lack of influence in clinical decision-making | Nursing autonomy  Shared decision making  Good collaboration between nurses and physicians | | | - Recognize the nursing domain alongside the medical domain in patient care processes. - Integrate clinical reasoning as part of structured workflows to strengthen trust and collaboration between nurses and physicians. - Utilize evidence-based practice to enhance care quality and support nursing leadership. - Foster a culture of continuous, structured interdisciplinary rounds discussing patient cases to drive team-based care improvement. - Promote a safe work environment for voicing concerns and making suggestions in patient care across disciplines. | |
| Limited career development opportunities | Career progression at all education levels  Diversity in tasks | | | - Ensure adequate staffing to facilitate career advancement opportunities, including protected time for nurses to engage in additional professional responsibilities. - Personalize career growth opportunities based on individual strengths and interests. - Enable nurses to pioneer in additional roles and support this development. - Provide horizontal career progression to broaden expertise within the organization. - Offer accessible professional development (e.g., structured case discussions). - Establish clear career pathways with role definitions, including combination roles. - Incorporate leadership training in nursing education and practice to enhance nurses’ ability to implement change. - Allow for specialization and expertise-driven differentiation within existing roles. - Promote job crafting to align roles with professional aspirations. - Create a work environment where additional roles and responsibilities are recognized equally as contributing to patient care. | |
| Poor work-life balance | | Flexible scheduling and autonomy over the schedule | - Adapt shift schedules to nurses’ individual biological rhythms. - Provide on-site childcare for irregular work hours. - Ensure adequate mental and physical recovery time in roster planning. - Offer flexible contract options, including small contracts (<24h). - Guarantee stable schedules for combination roles to prevent excessive workload fluctuations. - Properly incorporate additional staffing into rosters to prevent unnecessary restrictions. - Implement self-scheduling with minimal managerial interference. | |  |
| Low salary | | Financial and logistical support | - Offer salary structures that match professional development. - Provide discounted housing for healthcare professionals. - Enhance accessibility to the hospital by improving parking, bicycle facilities, and public transportation. - Offer financial incentives, such as improved reimbursement structures (free parking, public transport allowance) - Ensure meal provisions during evening and night shifts. - Address wage disparities by negotiating a nursing-specific collective labor agreement (CLA). - Develop a separate CLA to ensure that salary increases directly benefit essential healthcare personnel. | |  |
| Moral distress due to excessive workload and compromised patient safety | | Autonomy and control over practice | - Maintain adequate staffing levels with qualified personnel. - Implement nursing assistants as needed to alleviate the workload. - Foster autonomy and control over nursing practice to enhance job satisfaction. - Reduce unnecessary documentation for protocol-based interventions to improve workflow efficiency. - Allow nurses to define their clinical responsibilities while ensuring a reasonable administrative workload. - Enable nurses to apply clinical reasoning to adjust protocol to meet patient needs. - Provide access to up-to-date medical equipment and supplies. - Ensure a well-functioning^1^ ICT system with 24/7 support to maintain operational efficiency. - Offer access to updated protocols and guidelines. - Create an open culture where concerns and suggestions about patient care are voiced across disciplines. - Improve patient flow to ensure patients receive care in the appropriate setting. | |  |
| Workplace aggression and inappropriate behavior | | Safe work environment | - Ensure nurses receive appropriate resources and management backing for a safe working environment. - Implement strict policies against workplace aggression to build trust in organizational support. - Provide communication training for nurses to manage increasingly assertive patient populations. - Acknowledge individual preferences in handling difficult situations. | |  |
| Policy decisions do not align with nursing practice | | Involvement of nurses in policymaking | - Ensure staff are well-informed about the implications of policy choices. - Act on nurses' input to enhance engagement. - Establish strategic nursing roles within management, healthcare policy and governance to increase influence. | |  |
| Lack of recognition at the unit level | | Supportive leadership  Positive work culture | - Ensure supervisors serve as role models and remain informed on professional advancements. - Adopt a coaching-based leadership approach, encouraging nurses to find solutions. - Utilize individual expertise and strengths to optimize team composition. - Promote nursing leadership within the organization to enhance visibility and influence. - Recognize the impact of mentorship and encourage senior staff to model professional behavior. - Act upon nurses’ feedback to improve team morale. - Foster mutual respect in interdisciplinary collaborations. - Celebrate team successes. - Establish a trusting team dynamic with humor, mutual support, and open discussions. - Ensure sufficient onboarding time tailored to individual learning needs. | |  |
| Lack of recognition at the organizational level | | Valuing nursing expertise and autonomy | - Recognize nursing expertise and allow nurses to shape their roles by integrating their expertise. - Maintain transparency regarding organizational changes (e.g., mergers, policy shifts). - Provide 24/7 up-to-date equipment and materials. - Celebrate achievements within the organization. - Offer structured career progression, combination roles, and salary structures that match professional development. - Encourage open feedback and responsiveness to nurses’ input. - Create a welcoming work environment with financial and logistical support (see recommendations ‘low salary’). - Offer financial incentives for further education, such as specialized training (e.g. ethics) or additional roles beyond bedside care. | |  |
| Lack of societal recognition | | Visibility of the nursing profession | - Improve public perception of the nursing profession. - Develop a clear national vision on nursing. - Highlight autonomy and career-shaping opportunities for nurses. - Establish strategic nursing roles in healthcare policy and governance. - Address wage disparities by negotiating a nursing-specific collective labor agreement (CLA). - Develop a separate CLA to ensure that salary increases directly benefit essential healthcare personnel. | |  |

*^1^ Well-functioning refers to each nurse’s own perception of what it means to function well, and may vary from person to person.*
